# Mesenchymal stem cell-secreted prostaglandin E_2_ ameliorates acute liver failure via attenuation of cell death and regulation of macrophage polarization

**DOI:** 10.1186/s13287-020-02070-2

**Published:** 2021-01-07

**Authors:** Jinglin Wang, Yang Liu, Haoran Ding, Xiaolei Shi, Haozhen Ren

**Affiliations:** 1 Department of Hepatobiliary Surgery, Affiliated Drum Tower Hospital of Nanjing University Medical School, Nanjing, Jiangsu Province China; 2 Department of Hepatobiliary Surgery, Nanjing Drum Tower Hospital Clinical College of Nanjing Medical University, Nanjing, Jiangsu Province China; 3 Department of Hepatobiliary Surgery, Nanjing University of Chinese Medicine, Nanjing, Jiangsu Province China

**Keywords:** Acute liver failure, MSC, PGE_2_, mTOR, Macrophages

## Abstract

**Background:**

Acute liver failure (ALF) is an acute inflammatory liver disease with high mortality. Previous preclinical and clinical trials have confirmed that mesenchymal stem cell (MSC) is a promising therapeutic approach; however, the effect is not satisfied as the underlying molecular mechanisms of MSC in treating ALF remain unclear.

**Methods:**

MSC isolated from 4- to 6-week-old C57BL/6 mice were used to treat ALF. Histological and serological parameters were analyzed to evaluate the efficacy of MSC. We explored the molecular mechanism of MSC in the treatment of ALF by detecting liver inflammatory response and hepatocyte death.

**Results:**

In this study, we found that the therapeutic potential of MSC on ALF is dependent on the secretion of prostaglandin E_2_ (PGE_2_), a bioactive lipid. MSC-derived PGE_2_ inhibited TGF-β-activated kinase 1 (TAK1) signaling and NLRP3 inflammasome activation in liver macrophages to decrease the production of inflammatory cytokines. Meanwhile, macrophages in the liver could be induced to anti-inflammatory (M2) macrophages by MSC-derived PGE_2_ via STAT6 and mechanistic target of rapamycin (mTOR) signaling, which then promote inflammatory resolution and limit liver injury. Finally, administrating EP4 antagonist significantly ameliorated the therapeutic ability of MSC, which promoted liver inflammation and decreased M2 macrophages.

**Conclusions:**

Our results indicate that PGE_2_ might be a novel important mediator of MSC in treating ALF, which is through inhibiting the liver inflammatory response and hepatocyte death.

## Introduction

Acute liver failure (ALF) is a clinical syndrome with high mortality rate, and liver transplantation is the only effective way to cure ALF now [1]. Due to the shortage of organ donor resources, alternative treatments are needed urgently [2]. It has been shown that mesenchymal stem cell (MSC) not only rescued several animal models of liver injury, but that it showed an effective therapeutic potential in clinical trials, which is based on their ability to secrete various trophic factors [3]. Our previous study confirmed that MSC-derived PGE_2_ could promote hepatocyte proliferation in ALF; however, the role of MSC-derived PGE_2_ on liver inflammation still remains unclear.

Several studies confirmed the cell death and hepatic inflammation in the progression of ALF [4]. Inflammatory mediators of toxic liver injury include cytokines such as tumor necrosis factor (TNF), interleukin (IL)-1β, and reactive oxygen species (ROS) which accelerate liver injury [5]. Most of these inflammatory mediators were secreted by innate immune cells in the liver, including monocytes, macrophages, and dendritic cells (DC), which are activated by damage-associated molecular patterns (DAMPs) released by damaged hepatocytes. Liver macrophages hold central functions in initiating, perpetuating, and restricting inflammation. Macrophages can be classified into pro-inflammatory (M1) or anti-inflammatory (M2) macrophages [6]. M1 cells secrete large amounts of pro-inflammatory cytokines, such as TNF and IL-1β. M2 cells secrete immunosuppressive cytokines, like IL-10, which plays a vital role in the resolution of inflammation. Recent studies have confirmed the role of MSC on macrophage polarization. For example, the therapeutic ability of MSC on cartilage repair and sepsis was dependent on the induction of M2 macrophages [7, 8]. In various liver disease, the therapeutic ability of MSC also relies on macrophage polarization [9, 10]. However, the mechanism of MSC on macrophage polarization remains unclear.

Sterile inflammation, which is defined as an inflammatory response triggered by damage mediated by noninfectious sources, plays a major role in ALF [4]. The nucleotide-binding and oligomerization domain-like receptor 3 (NLRP3) inflammasome is the main kind of sterile inflammation, which contains three parts: NLRP3, apoptosis-associated speck-like protein (ASC), and pro-caspase 1. The inflammasome is activated by DAMPs or pathogen-associated molecular patterns (PAMPs), which contribute to the maturation and secretion of IL-1β and IL-18 [11]. Recent studies confirmed that inflammasome activation contributes to the progression of various liver disorders, like viral fulminant hepatitis and nonalcoholic fatty liver disease [12]. Therefore, targeting the NLRP3 inflammasome might be an effective strategy for reducing liver inflammation.

In this study, we focused on the inflammatory response in ALF. We confirmed that MSC could attenuate liver inflammation and NLRP3 inflammasome activation; meanwhile, it promoted M2 macrophages to resolve inflammation, thus to cure ALF.

## Materials and methods

### Isolation of mouse bone marrow MSC

MSCs were isolated from 4- to 6-week-old C57BL/6 mice as we previously described [13]. We used flow cytometry (BD FACSAria II; BD Biosciences, San Jose, CA, USA) to identify the MSCs; the antibodies used were against mice antigens CD29, CD34, CD44, CD45, and CD90 (BD Biosciences). Positive cells were counted and compared to the signals of corresponding immunoglobulin isotypes. Mediums were supplemented with 10% fetal calf serum (Sciencell, San Diego, CA, USA), 100 U/ml penicillin, and 100 g/ml streptomycin. To deplete or overexpress COX2 in MSC to inhibit or increase the synthesis and secretion of PGE_2_, the short hairpin RNA against COX2 or COX2 gene lentivirus (GeneChem, Shanghai, China) was transfected to MSCs following the manufacturer’s instructions [named MSC-COX2(−) and MSC-COX2(+)].

For MSC, MSC-COX2(−)- or MSC-COX2 (+)-conditioned medium, MSCs were maintained in serum-free medium for 48 h at 37 °C in a humidified atmosphere containing 5% CO_2_. Conditioned medium was harvested, clarified by centrifugation, and frozen at − 80 °C until use.

### Animal models

Male C57BL/6J wild type mice (6~8 weeks old) were obtained from the Animal Core Facility of Nanjing Medical University. Animals were bred in a pathogen-free facility. Mice were randomly divided into five groups: (1) control group (Ctrl): mice were intraperitoneally (i.p.) injected with PBS only; (2) ALF group (ALF): mice simultaneously received 600 mg/kg D-galactosamine (D-Gal) (Sigma-Aldrich, St. Louis, MO, USA) and 100 μg/kg LPS (Sigma-Aldrich) dissolved in PBS by i.p. injection; (3–5) cell transplantation groups: mice received 600 mg/kg D-Gal and 100 μg/kg LPS via i.p. injection, and then 2 × 10^6^ MSC, MSC-COX2(+), or MSC-COX2(−) were injected via tail vein after 6 h, namely the MSC group, MSC-COX2(+) group, or MSC-COX2(−) group, respectively. For TAK1 inhibition, we administrated the specific TAK1 inhibitor 5z-7-ox (5 mg/kg, Sigma) to mice 1 h before LPS/D-Gal administration. To inhibit the EP4 receptor of PGE_2_, we administered EP4-specific antagonist (GW627368X, 20 mg/kg, Cayman Chemical, Ann Arbor, MI, USA) 1 h before LPS/D-Gal treatment and every 24 h following MSC infusion. Blood and liver tissues were collected at the indicated time for further analysis.

### Bone marrow-derived macrophage (BMDM) isolation and culture

BMDMs were prepared and cultured as described [14]. In brief, BM cells were flushed from femurs and tibias of mice and dispersed mechanically. Cell suspensions were filtered through a 200-mesh filter, and the remaining cells were collected by centrifugation at 300×*g* for 4 min. After centrifugation, the cells were resuspended and cultured in RIP 1640 supplemented with 10% FBS and macrophage colony-stimulating factor (M-CSF, 20 ng/ml, eBioscience) for 7 days.

For inflammasome activation, BMDMs were treated with LPS (100 ng/ml) for 4 h, followed by 30-min treatment with PGE_2_ (0.1 μM) or MSC, followed by stimulation with nigericin (4 μM for 1 h, Cayman Chemical). EP4 antagonist was added 30 min prior to treatment with PGE_2_ or MSC. For macrophage polarization, BMDMs were stimulated with PGE_2_ or MSC for 24 h.

### Real-time quantitative PCR and western blotting

Total RNA or protein for liver tissues and cells were extracted as previously described [13]. The primer sequences and primary antibodies are listed in Supplementary Table S1 and S2.

### Small interfering RNA and transfection

siRNA-specific EP4 as well as control siRNAs were obtained from RiboBio (GuangZhou, China). The detailed sequences of siRNAs are listed in Supplementary Table S3. Transfection with siRNAs was completed using riboFECT™ CP (RiboBio) according to the manufacturer’s instruction.

### Immunofluorescence and HE analyses

HE staining analysis of the liver sections was conducted as described previously [13]. The images were captured by a Nikon microscope (Japan). Other sets of sections were dewaxed and subjected for immunofluorescence labelling of NLRP3 (1:200, Adipogen Life, San Diego, USA) and F4/80 (1:200, Abcam, Cambridge, UK). Sections were incubated with the primary antibodies at the same working dilutions as noted above overnight at 4 °C, followed by incubation with goat anti-rabbit IgG H&L (Alexa Fluor® 488) (1:500, Abcam) or Goat Anti-Rat IgG H&L (1:500, Alexa Fluor® 594) for 1 h. Nuclei were counterstained with 4′,6-diamidino-2-phenylindole (DAPI, KeyGen BioTECH, Nanjing, China), and images were captured using fluorescence microscopy (Olympus, Japan).

### Measurement of serum alanine aminotransferase (ALT) and aspartate aminotransferase (AST)

Serum ALT and AST were determined using an automatic biochemical analyzer (Olympus, Tokyo, Japan) in the Affiliated Drum Tower Hospital of Nanjing University Medical School.

### TUNEL and PI assay

Cell apoptosis and death in the liver were measured using the one-step TUNEL Apoptosis Assay kit and propidium iodide (PI) staining (Beyotime Biotechnology, Shanghai, China) according to the manufacturer’s instructions.

### IL-1β and IL-10 analysis

IL-1β levels in serum or cell supernatant and IL-10 levels in serum were detected by an enzyme-linked immunosorbent assay (ELISA) kit (Invitrogen, Thermo Scientific, Vienna, Australia).

### Caspase 1 activity

Caspase-1 activity in liver tissues was measured by Caspase 1 Activity Assay Kit (Beyotime Biotechnology) according to the manufacturer’s instructions.

### Statistical analysis

Data were expressed as means ± SD. Differences between groups were assessed by a two-tailed *t* test assuming unequal variance. Five to 8 mice/group were analyzed using Prism software (GraphPad). *p* values less than 0.05 were considered as statistically significant.

## Results

### MSC protects against ALF via PGE_2_

**Fig. 1:**
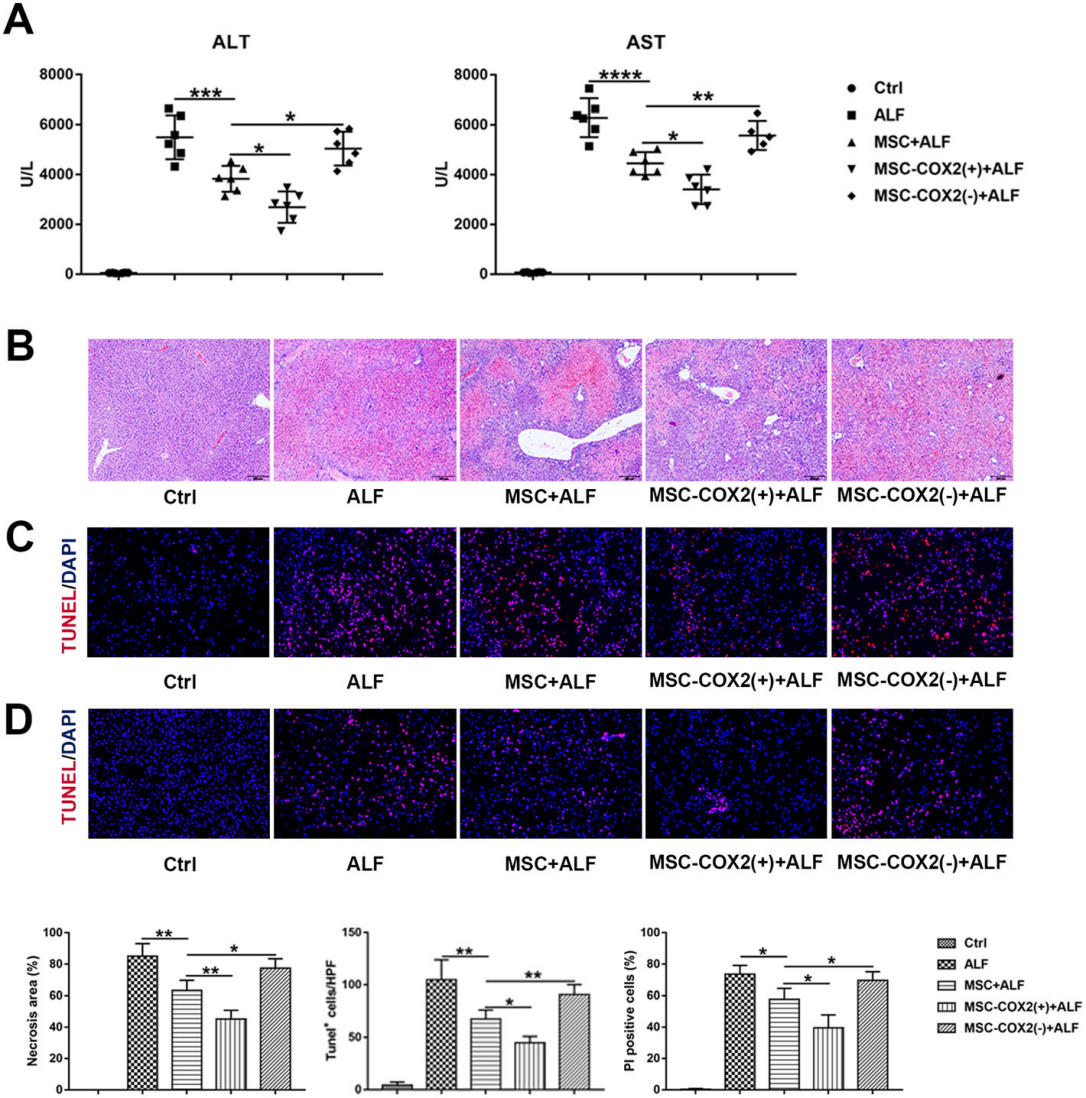
MSC protects LPS/D-Gal-induced liver injury via PGE_2_. **a** Serum levels of ALT and AST in each group (*n* = 6). **b** Representative HE-stained liver sections from each group and quantitation of the necrosis area in each group (*n* = 4). **c** Representative images of TUNEL-stained liver sections from each group and the number of TUNEL-positive cells in each group (*n* = 4). **d** Representative images of PI staining to indicate necrosis cells and the number of PI-positive cells in each group (*n* = 4) (*p < 0.05, **p < 0.01, ***p < 0.001, ****p < 0.0001)

Paralyzed with our previous study [13], MSC protected against ALF through PGE_2_, as revealed by plasma levels of ALT and AST (Fig. 1a) and liver damage in HE staining (Fig. 1b). TUNEL and PI staining confirmed the hepatocyte necrosis and apoptosis were decreased when infused with MSC, especially in the MSC-COX2(+) group; however, MSC-COX2(−) failed to decrease hepatocyte necrosis and apoptosis (Fig. 1c, d). Taken together, MSC protected hepatocyte death against ALF via PGE_2_.

### MSC ameliorates inflammation in ALF

**Fig. 2:**
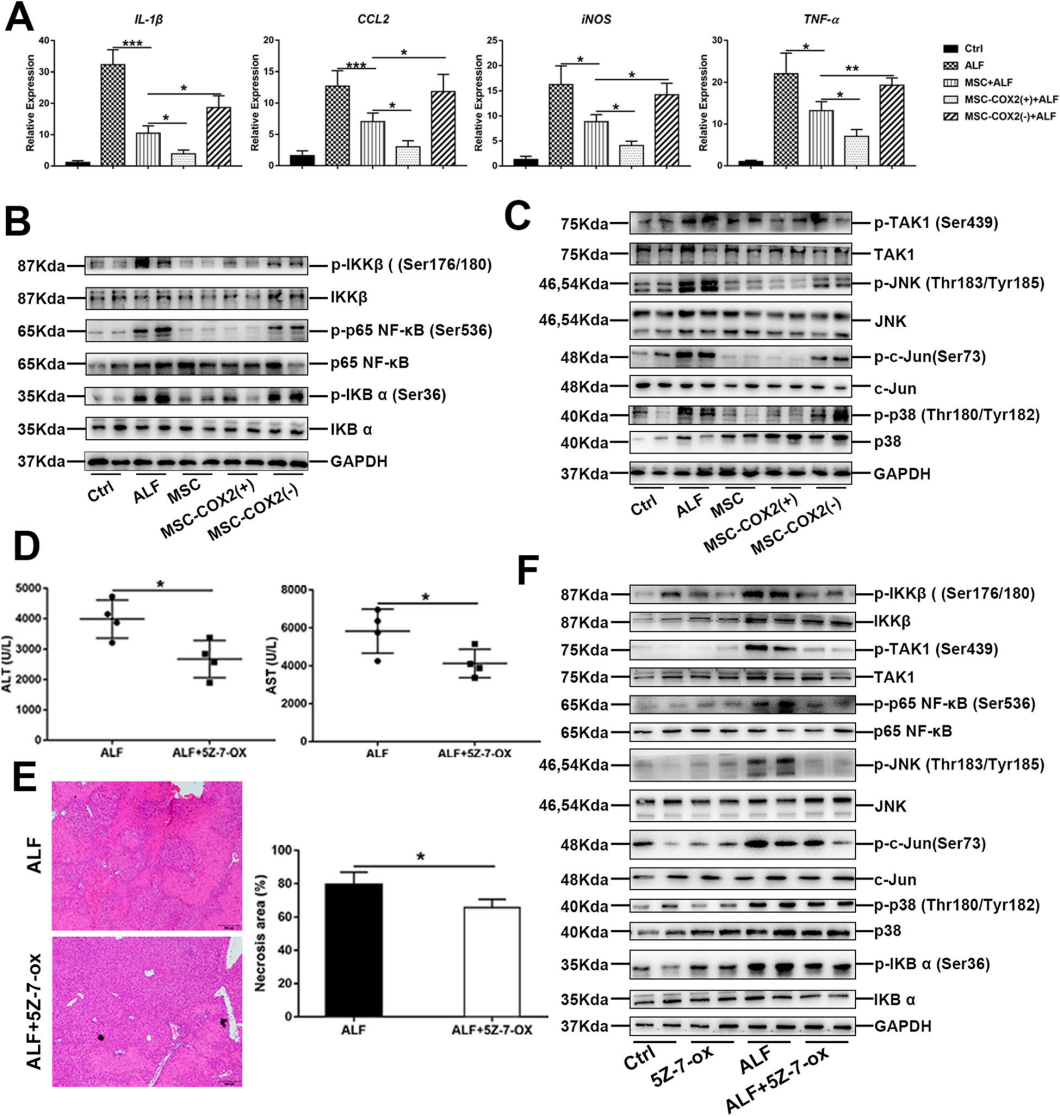
MSC-derived PGE_2_ restrains inflammatory responses in the liver during LPS/D-Gal-induced ALF. **a** The mRNA levels of pro-inflammatory cytokines (*IL-1β*, *CCL2*, *iNOS*, *TNF-α*) in the liver in each group (*n* = 4). **b** The protein expression levels of NF-κB signaling in each group. **c** The protein expression levels of TAK1 signaling in each group. **d** Serum levels of ALT and AST in mice pretreated with TAK1 inhibitor, 5Z-7-ox (*n* = 4). **e** Representative HE-stained liver sections and quantitation of necrosis area in each group (*n* = 4). **f** Protein levels of TAK1 and NF-κB signaling in each group (*p < 0.05, **p < 0.01, ***p < 0.001)

Liver inflammation is associated with hepatocyte damage and death; therefore, we examined the inflammatory response in the liver after MSC infusion. Notably, mRNA expression levels of inflammatory cytokines and chemokines, including *CCL2*, *IL-1β*, *iNOS*, and *TNF-α* were significantly inhibited in the MSC group, and in the MSC-COX2(+) group, the levels of these cytokines decreased more obviously. By contrast, the MSC-COX2(−) group, which inhibited secretion of PGE_2_ from MSC, failed to decrease these inflammatory cytokines (Fig. 2a). NF-κB signaling plays a great role in inflammation [15]. We demonstrated that LPS/D-Gal-induced ALF significantly activated NF-κB signaling in the liver, as the increased expression of the phosphorylation of IKK-β and P65 (p-IKK-β and p-P65) (Fig. 2b). The MSC or MSC-COX2(+) group exhibited an ameliorated phenotype, while MSC-COX2(−) did not show this phenotype (Fig. 2b). Next, we aimed to explore the mechanism of MSC on NF-κB signaling inhibition. TGF-β-activated kinase 1 (TAK1) is an intracellular hub molecule that regulates NF-κB signaling pathways which plays a key role in cell survival and death [16]. Our results demonstrated that TAK1 signaling was inhibited after MSC infusion, as the expression of the phosphorylation of TAK1 (p-TAK1), JNK (p-JNK), P38 (p-P38), and c-Jun (p-c-Jun) were significantly reduced in the MSC or MSC-COX2(+) group, while these TAK1 substrates still expressed high in the MSC-COX2(−) group (Fig. 2c).

To confirm the role of TAK1 in ALF, we further used 5Z-7-ox, a specific TAK1 inhibitor, to inhibit TAK1 before LPS/D-Gal administration. Notably, TAK1 inhibition protected against liver damage, shown by normalized serum ALT and AST and reduced necrosis on the histological analysis (Fig. 2d, e). Treatment of 5Z-7-ox significantly suppressed TAK1 activation and reduced activation of its downstream signaling pathways, including p-JNK, p-P38, p-c-Jun, and NF-κB signaling (Fig. 2e). Collectively, these observations suggested that MSC-derived PGE_2_ protected ALF through suppressing liver inflammation in a TAK1-NF-κB pathway.

### MSC inhibits inflammasome activation of macrophages in ALF

**Fig. 3:**
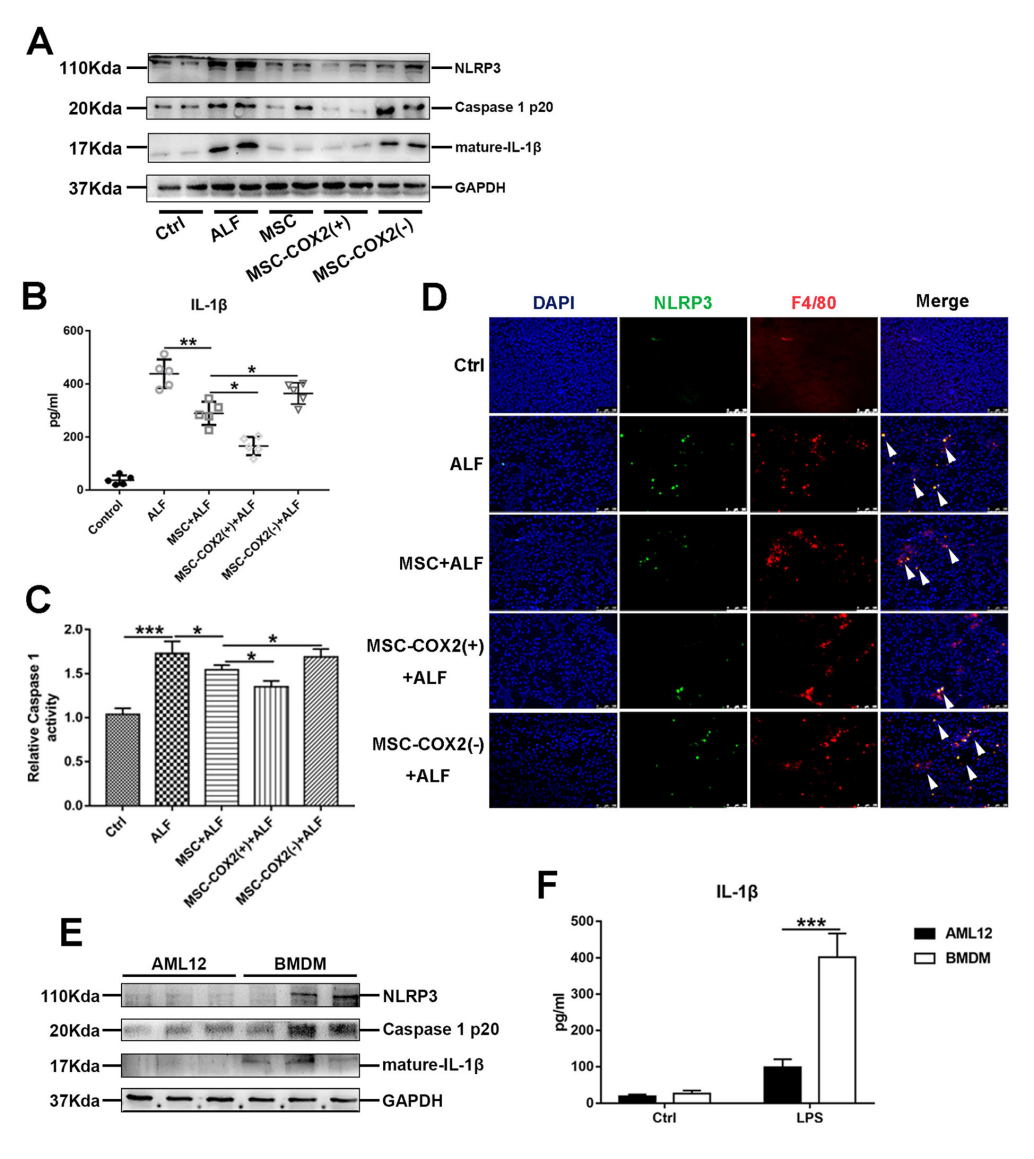
MSC-derived PGE_2_ inhibits NLPR3 inflammasome activation in liver macrophages. **a** Protein levels of NLRP3, Caspase 1 p20, and mature-IL-1β in each group. **b** Serum levels of IL-1β in each group (*n* = 6). **c** Measurement of caspase-1 enzymatic activity in the liver of each group (*n* = 4). **d** Representative immunofluorescence staining of co-localization of NLRP3 with F4/80 in the liver of each group. Blue: DAPI; green: NLRP3; red: F4/80. White arrows in the Merge images indicated co-localization. **e** Protein levels of NLRP3 inflammasome pathway in hepatocyte cell line AML12 and BMDM treated with 1 μg/ml LPS for 24 h. **f** The levels of IL-1β in supernatants of hepatocyte cell line AML12 and BMDM treated with 1 μg/ml LPS for 24 h (*n* = 3) (*p < 0.05, **p < 0.01, ***p < 0.001)

Previous studies have confirmed the role of inflammasome activation in LPS/D-Gal-induced liver injury, which accelerated liver inflammation [17]. Next, we explored whether MSC infusion could inhibit NLRP3 inflammasome activation in the liver. The protein levels of NLRP3, caspase1 p20, and mature-IL-1β were decreased in the MSC or MSC-COX2(+) group (Fig. 3a). In parallel, serum concentrations of IL-1β and the activity of caspase 1 enzyme in liver tissues confirmed the inhibition of NLRP3 inflammasome by MSC-derived PGE_2_ (Fig. 3b, c). However, MSC-COX2(−) failed to inhibit NLRP3 inflammasome activation in liver tissues. In order to identify the cell population responsible for increased production of NLRP3 inflammasome, the double immunohistochemistry staining was performed. The results demonstrated that NLRP3 was mostly activated in macrophages in the liver and MSC or MSC-COX2(+) could inhibit NLRP3 activation in liver macrophages (Fig. 3d). To confirm this result, we treated mouse hepatocyte cell line AML12 or BMDM with 1 μg/ml LPS for 24 h in vitro. As showed in Fig. 3e and f, the activation of NLRP3 and levels of IL-1β secretion was much higher in BMDM, which indicated that macrophages were more sensitive to DAMPs than hepatocytes. Collectively, our results indicated that MSC-derived PGE_2_ inhibited liver macrophage inflammasome activation to protect against liver injury.

**Fig. 4:**
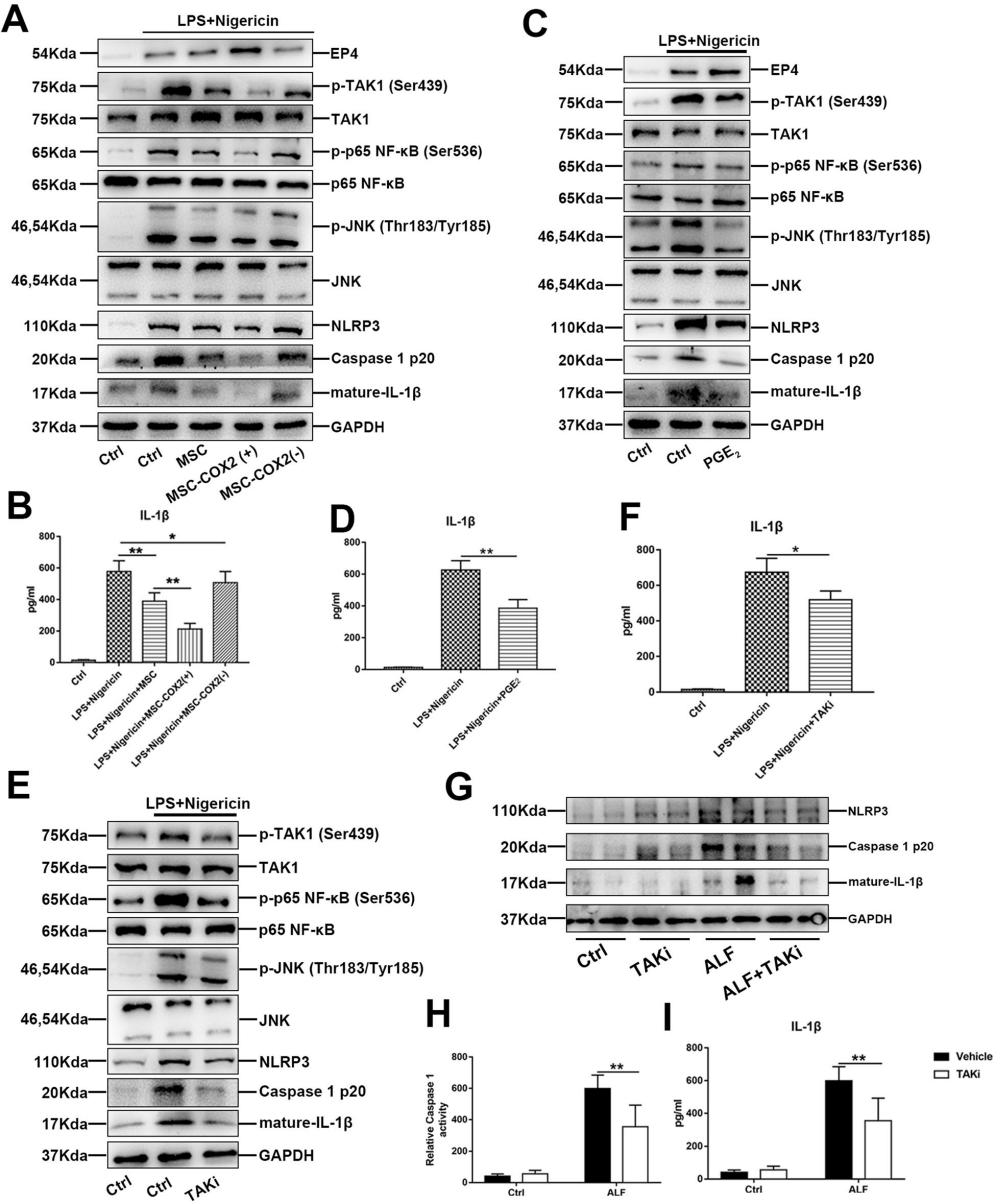
MSC-derived PGE_2_ inhibits NLRP3 inflammasome activation via TAK1. **a** The protein expression levels of NLRP3 inflammasome and TAK1 signaling in BMDM following indicated condition. **b** The levels of IL-1β in each supernatant (*n* = 3). **c** The protein expression levels of NLRP3 inflammasome and TAK1 signaling in BMDM treated with exogenous PGE_2_. **d** The levels of IL-1β in cell supernatant treated with exogenous PGE_2_ (*n* = 3). **e** Effects of TAK1 inhibitor, 5Z-7-ox (TAKi), on NLRP3 inflammasome activation in BMDM. **f** The levels of IL-1β in cell supernatant treated with TAKi (*n* = 3). **g** Expression of NLRP3 inflammasome signaling in the liver pretreated with TAKi. **h** Measurement of caspase-1 enzymatic activity in the liver of each group (*n* = 4). **i** Serum levels of IL-1β in each group (*n* = 4) (*p < 0.05, **p < 0.01)

To confirm the role of MSC on NLRP3 inhibition, we treated BMDM with LPS and nigericin, a typical inducer of NLPR3. MSC or MSC-COX2(+)-conditioned medium significantly inhibited NLPR3 activation and IL-1β secretion (Fig. 4a, b), while MSC-COX2(−)-conditioned medium did not show this phenomenon. In parallel, exogenous PGE_2_ exhibited the same effects on NLRP3 (Fig. 4c, d). Previous studies have confirmed the role of NF-κB in NLRP3 inflammasome activation [18]; thus, we explored whether MSC inhibited NLRP3 through TAK1-NF-κB. Our results showed that MSC-conditioned medium or PGE_2_ could inhibit TAK1 and NF-κB activation in BMDM induced by LPS and nigericin (Fig. 4a, c). Meanwhile, TAK1 inhibitor could also inhibit NLRP3 activation in BMDM (Fig. 4e, f). In vivo study confirmed the effects of TAK1 inhibitor on NLRP3 in the ALF model (Fig. 4g, h). Overall, our results indicated that MSC-derived PGE_2_ could inhibit NLPR3 inflammasome activation of liver macrophages through TAK1-NF-κB pathway.

### MSC induces M2 macrophage polarization in ALF

**Fig. 5:**
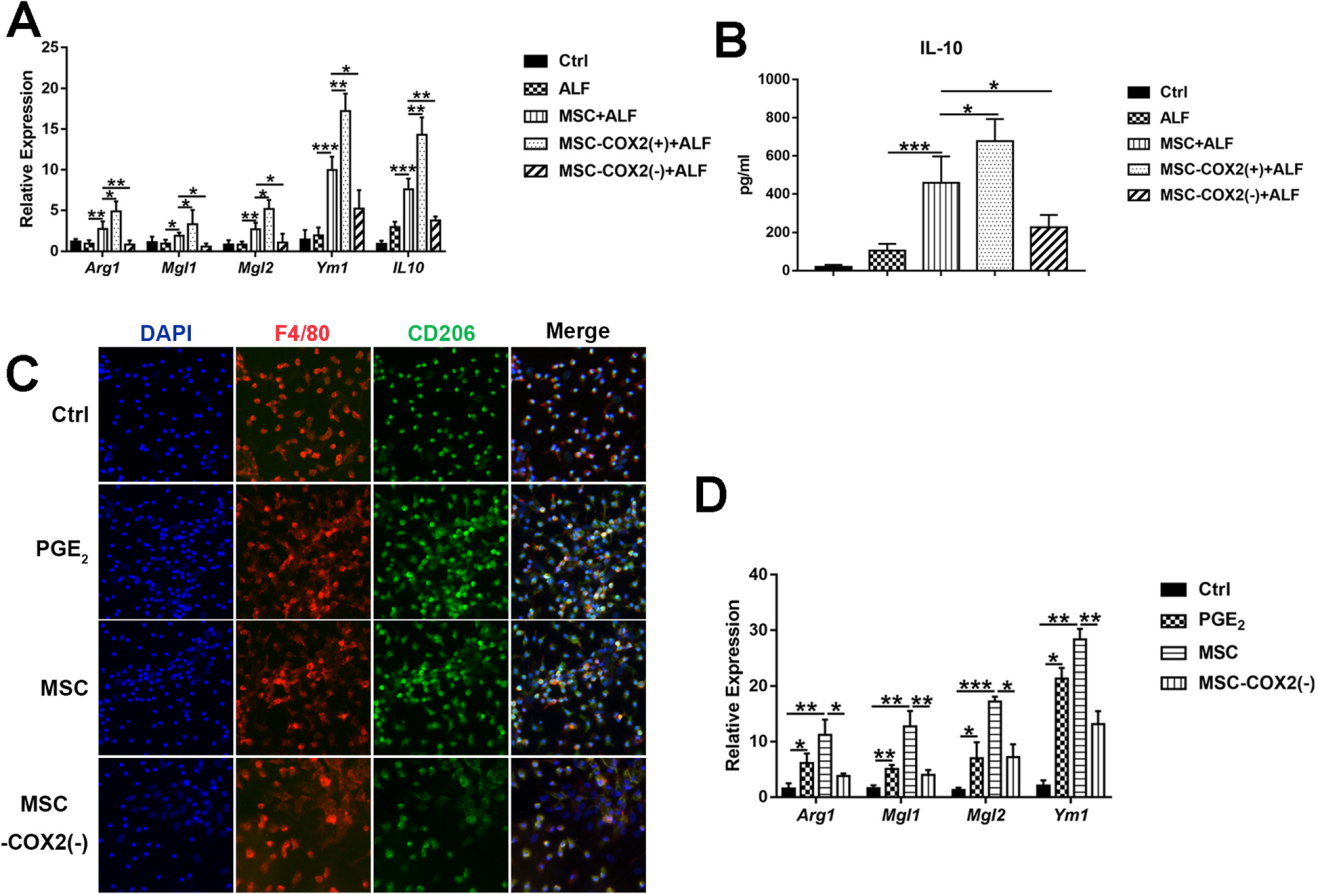
MSC-derived PGE_2_ promotes an M2 macrophage phenotype in the liver. **a** The mRNA levels of M2 markers (*Arg1*, *Mgl1*, *Mgl2*, *Ym1*, and *IL-10*) in the liver of each group (*n* = 4). **b** Serum levels of IL-10 in each group (*n* = 4). **c** Representative immunofluorescence staining of M2 marker, CD206, and F4/80 in BMDM following indicated condition. **d** The mRNA levels of M2 markers in BMDM following indicated condition (*n* = 3) (*p < 0.05, **p < 0.01, ***p < 0.001)

Mouse models of acute liver injury have revealed the role of macrophages in accelerating damage through cytokines and chemokines releasing. However, liver macrophages are plastic and adapt their phenotype to promote tissue repair according to signals derived from the hepatic microenvironment [6]. Several studies confirmed the role of MSC on macrophage polarization in different models [19, 20]. Thus, we tended to explore whether MSC could induce M2 macrophages in ALF to promote inflammation resolution. We found that gene expression associated with M2-like (*Arg1*, *Mgl1*, *Mgl2*, and *Ym1*) macrophages were increased in the MSC and MSC-COX2(+) groups (Fig. 5a); meanwhile, the serum levels of IL-10, which is produced by M2 macrophages, were also increased in the MSC and MSC-COX2(+) groups (Fig. 5b). However, MSC-COX2(−) failed to induce M2 macrophages. In vitro studies confirmed the MSC-derived PGE_2_ on macrophage polarization, as the expression of M2 markers and numbers of CD206-positive cells of BMDM treated with MSC-conditioned medium or PGE_2_ were increased (Fig. 5c, d). Taken together, our results demonstrated that MSC induced M2 macrophages to resolute inflammation in ALF through PGE_2_.

**Fig. 6:**
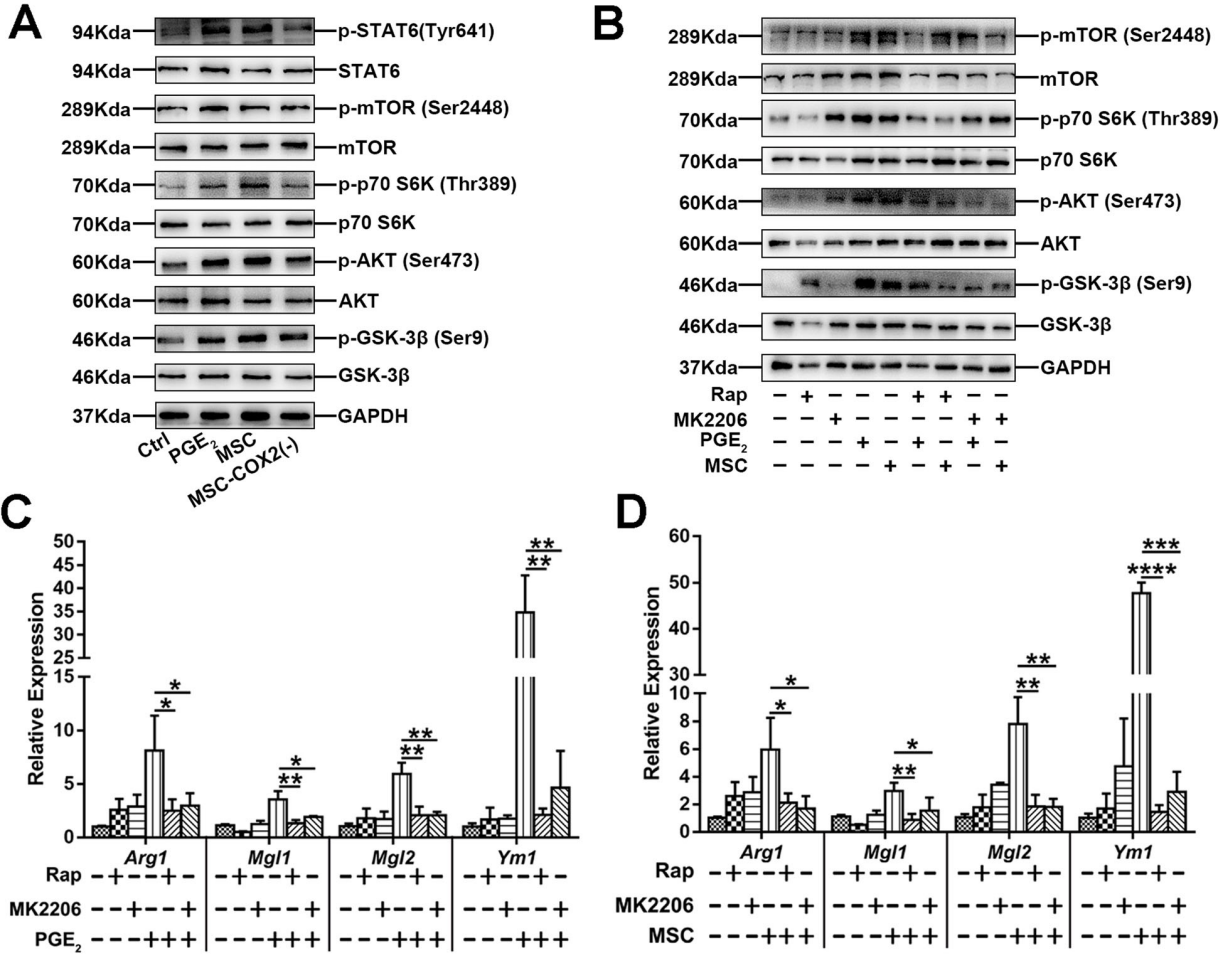
MSC-derived PGE_2_ promotes M2 macrophage through STAT6 and mTOR signaling. **a** Protein levels of STAT6 and mTOR signaling in BMDM following indicated condition. **b** Protein levels of mTOR signaling in BMDM treated with mTOR inhibitor (rapamycin, Rap) and AKT inhibitor (MK-2206) following indicated condition. **c** The mRNA levels of M2 markers (*Arg1*, *Mgl1*, *Mgl2*, and *Ym1*) in BMDM treated with Rap following indicated condition (*n* = 3). **d** The mRNA levels of M2 markers (*Arg1*, *Mgl1*, *Mgl2*, and *Ym1*) in BMDM treated with MK-2206 following indicated condition (*n* = 3) (*p < 0.05, **p < 0.01, ***p < 0.001, ****p < 0.0001)

Next, we investigated the mechanism of MSC-derived PGE_2_ on macrophage polarization. Western blotting results showed that not only the classical M2 pathway, STAT6, was activated when treated with MSC or PGE_2_, but also resulted in mTOR activation, as increased phosphorylation of the mTOR substrates mTOR, S6K, AKT, and GSK-3β (Fig. 6a), which correlated with previous studies [21]. In contrast, MSC-COX2(−)-conditioned medium failed to activate mTOR and STAT6 pathway (Fig. 6a). Treatment of BMDM with mTOR inhibitor, rapamycin (Rap), or AKT inhibitor, MK-2206, confirmed the role of mTOR signaling in MSC-derived PGE_2_ on M2 macrophage polarization, as the mRNA levels of M2 markers were significantly increased after administration of Rap or MK-2206 (Fig. 6b–d). Taken together, our results indicated that MSC-derived PGE_2_ induced M2 macrophages through mTOR and classical STAT6 pathway.

### MSC protects against liver inflammation via PGE_2_ receptor (EP) 4

**Fig. 7:**
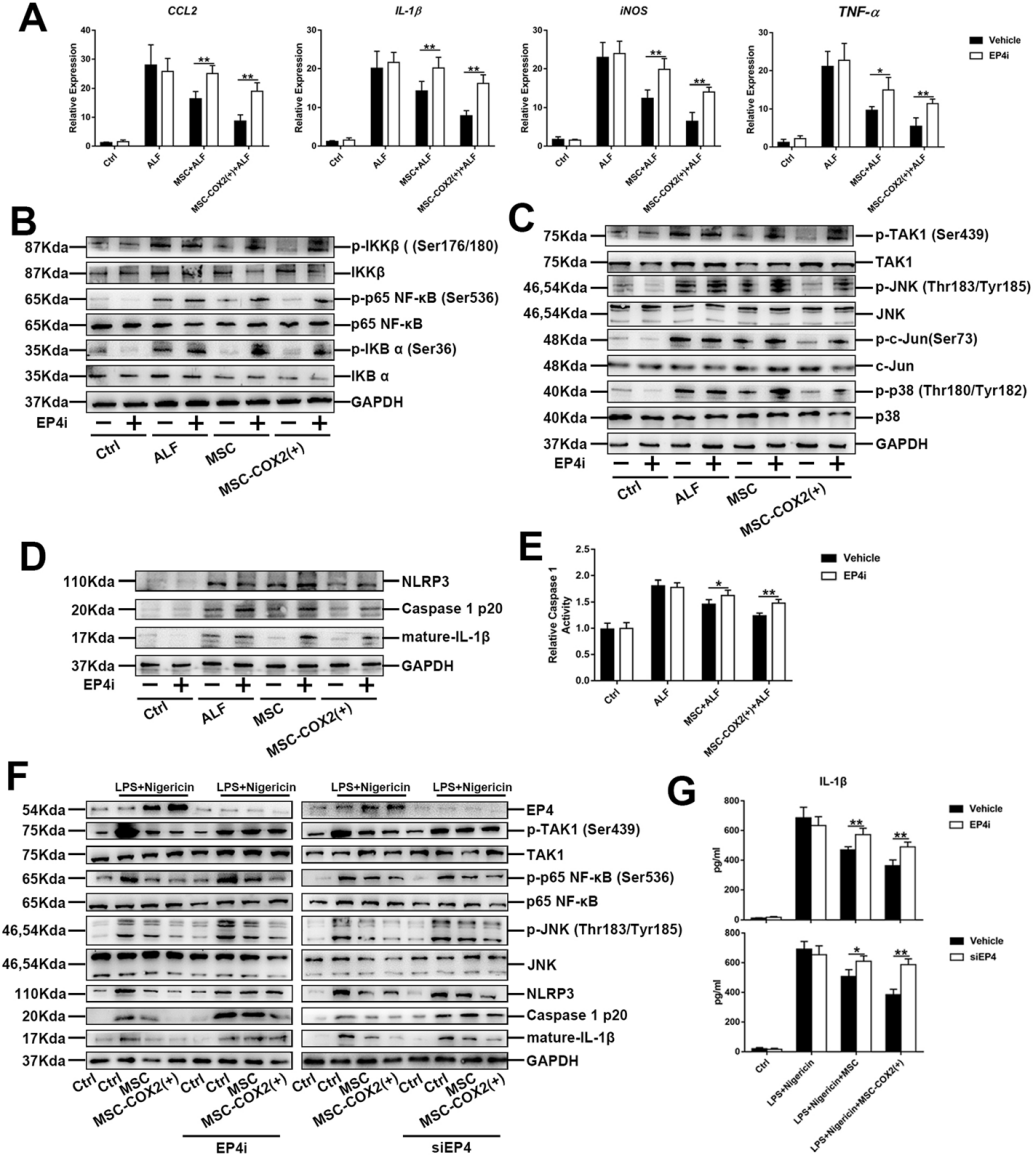
MSC-derived PGE_2_ protects against liver inflammation via EP4. **a** The mRNA levels of inflammatory cytokines (*IL-1β*, *CCL2*, *iNOS*, *TNF-α*) in the liver from each group pretreated with EP4 inhibitor (EP4i) (*n* = 4). **b** The protein expression levels of NF-κB signaling in each group pretreated with EP4i. **c** The protein expression levels of TAK1 signaling in each group pretreated with EP4i. **d** The protein expression levels of NLRP3 inflammasome signaling in each group pretreated with EP4i. **e** Measurement of caspase-1 enzymatic activity in the liver of each group pretreated with EP4i (*n* = 4). **f** Protein levels of NLRP3 inflammasome and TAK1 signaling in BMDM pretreated with EP4i or knocking down of EP4 via siRNA. **g** The levels of IL-1β in supernatants of BMDM pretreated with EP4i or knocking down of EP4 via siRNA (*p < 0.05, **p < 0.01)

Our previous studies showed that MSC-derived PGE_2_ promoted hepatocyte proliferation through EP4 [13]; we explored whether EP4 also played a role in liver inflammation resolution. We treated mice with EP4 inhibitor (EP4i), GW627368X. The mRNA levels of inflammatory cytokines increased when treated with EP4i (Fig. 7a). Western blot results confirmed that inhibition of TAK1 and NF-κB signaling by MSC was also mediated by EP4, as the expression of TAK1 and NF-κB signaling substrates was higher when EP4i was administered (Fig. 7b, c). Meanwhile, the expression of NLRP3 and the activity of caspase 1 enzyme were also higher when EP4i was administered, even in the MSC-COX2(+) group (Fig. 7d, e). In vitro studies using EP4i or siRNA to inhibit or deplete EP4 in BMDM, the western blot and IL-1β secretion confirmed the role of EP4 on NLRP3 inflammasome inhibition (Fig. 7f, g). Taken together, these results showed that MSC-derived PGE_2_ ameliorated liver inflammation through EP4.

### EP4 mediates MSC-derived PGE_2_ on macrophage polarization

**Fig. 8:**
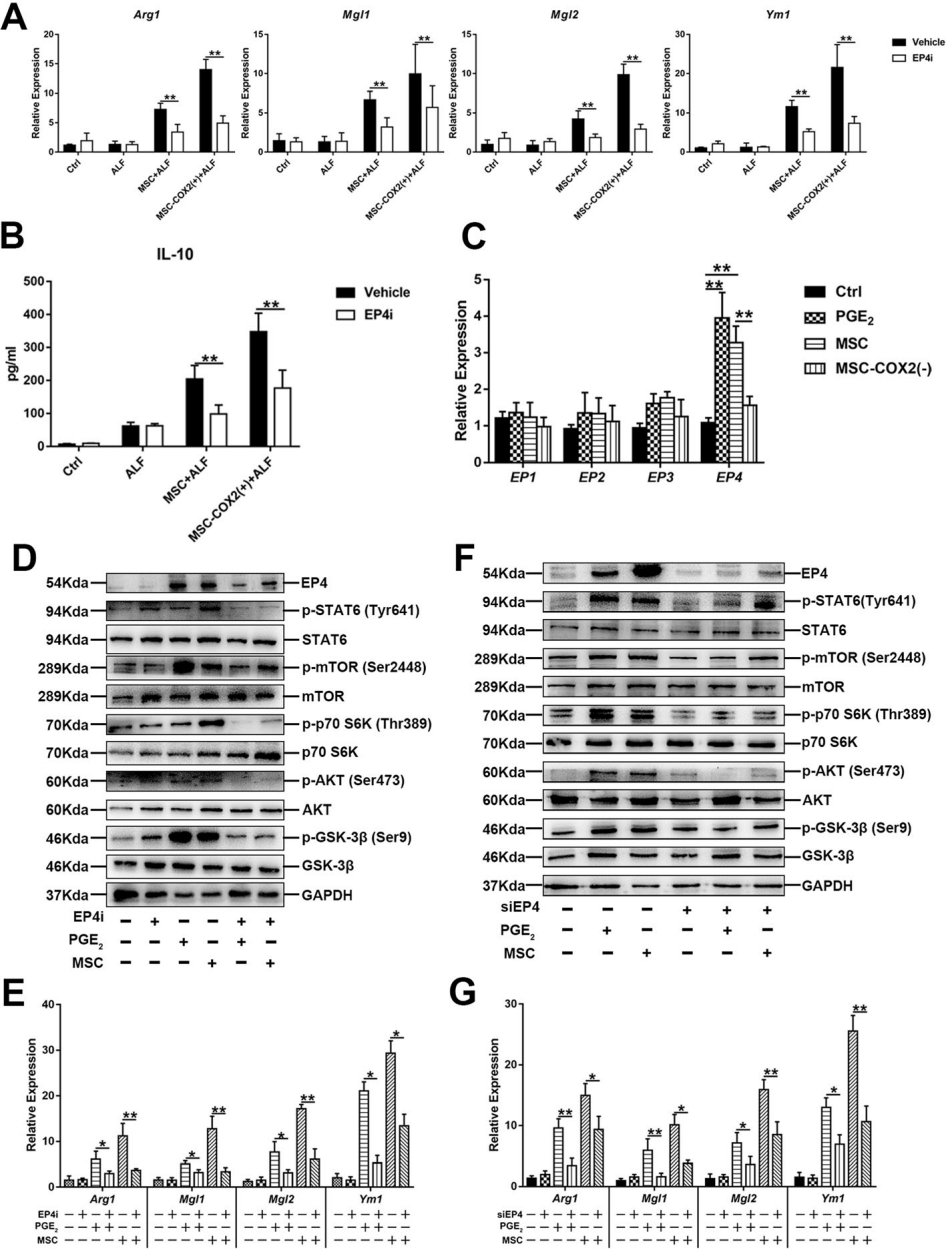
M2 macrophage polarization depends on EP4. **a** The mRNA levels of M2 markers (*Arg1*, *Mgl1*, *Mgl2*, and *Ym1*) in the liver of each group pretreated with EP4i (*n* = 4). **b** Serum levels of IL-10 in each group (*n* = 4). **c** The mRNA levels of (*EP1*, *EP2*, *EP3*, and *EP4*) in BMDM under indicated condition (*n* = 3). **d** Protein levels of STAT6 and mTOR signaling in BMDM treated with EP4i. **e** The mRNA levels of M2 markers (*Arg1*, *Mgl1*, *Mgl2*, and *Ym1*) in BMDM treated with EP4i (*n* = 3). **f** Protein levels of STAT6 and mTOR signaling in BMDM knocked down EP4 via siRNA. **g** The mRNA levels of M2 markers (*Arg1*, *Mgl1*, *Mgl2*, and *Ym1*) in BMDM knocked down EP4 via siRNA (*n* = 3) (*p < 0.05, **p < 0.01)

Next, we explored whether M2 macrophage polarization was also mediated by EP4. The mRNA levels of M2 macrophage markers and serum levels of IL-10 were also inhibited when treated with EP4i (Fig. 8a, b). And mRNA levels of PGE_2_ receptors (EP1-EP4) in BMDM confirmed the role of EP4 on M2 macrophage polarization (Fig. 8c). EP4i or siRNA treatment demonstrated the role of EP4 in macrophage polarization, as the mRNA levels of M2 markers were decreased when treated with EP4i or siRNA (Fig. 8e, g). Meanwhile, the protein levels of STAT6 and mTOR signaling substrates confirmed the role of EP4 on STAT6 and mTOR signaling (Fig. 8d, f). Overall, these results demonstrated that EP4 played a major role in MSC-derived PGE_2_ on macrophage polarization.

## Discussion

Cell death and inflammation play great roles in the progression of ALF [4]. Accordingly, we speculated that reduced cell death and hepatic inflammation would be beneficial for the prevention and treatment of ALF. As described previously, MSC could regulate these important pro-inflammatory cytokines via a variety of effector mechanisms [22]. In our study, we clarified that MSC-derived PGE_2_ reduced hepatocyte death and liver inflammation to attenuate ALF. Meanwhile, MSC-derived PGE_2_ also modulated macrophage polarization to promote inflammation resolution in ALF.

PGE_2_ is a bioactive lipid which is catalyzed by the COX2 from arachidonic acid. PGE_2_ exerts a wild range of biological effects associated with inflammation and cancer [23]. Several studies confirmed that PGE_2_ promoted the resolution of inflammation and tissue repair. In a concanavalin A (ConA)-induced liver injury model, depleted COX2 in mice accelerated liver injury [24]. Hepatocyte COX2 expression protected against liver ischemia-reperfusion injury (IRI); meanwhile, in patients who underwent liver transplantation, there was a significant positive correlation of plasma PGE_2_ levels and graft function [25]. These results indicated that PGE_2_ might have great therapeutic potentials in treating inflammatory liver diseases. In this study, we demonstrated the therapeutic role of MSC secreted PGE_2_ on LPS/D-Gal-induced ALF based on its anti-apoptosis and anti-inflammation ability.

We confirmed that MSC-derived PGE_2_ reduced pro-inflammatory cytokine production in the liver through inhibiting TAK1-NF-κB signaling, which is important in regulating the expression of pro-inflammatory cytokines. TAK1 is a central regulator of cell death and inflammation through activating downstream effectors such as NF-κB and mitogen-activated protein kinases (MAPKs) [16]. Recent studies have demonstrated the role of TAK1 in the progression of various liver diseases, like liver IRI, nonalcoholic steatohepatitis (NASH), and hepatocellular carcinoma [26–28]. In our study, we found that MSC infusion could decrease the expression of TAK1 downstream substrates and NF-κB, thus alleviating liver inflammation. In parallel, administration of the TAK1-specific inhibitor, 5Z-7-ox, could also protect against ALF in vivo.

Inflammasome activation has been identified as a major contributor to hepatocyte damage in ALF [29]. Our results showed that administration of MSC, especially MSC-COX2(+), significantly suppressed LPS/D-Gal-induced inflammasome activation, while MSC-COX2(−) failed to show this phenomenon, which demonstrated the role of PGE_2_ on NLRP3 inflammasome inhibition. Next, we confirmed that macrophages in the liver are the main source of NLRP3, which then accelerated liver inflammatory response. A previous study has showed that TAK1 restricted NLRP3 activation and NF-κB was important for NLRP3 activation [18, 30]. We found that mice pretreated with 5Z-7-ox significantly inhibited NLRP3 inflammasome activation, which correlated with the in vitro model results, showing the role of TAK1 on NLRP3 inflammation activation. These results indicated that MSC-derived PGE_2_ restricted macrophage NLRP3 activation through TAK1.

Liver macrophages hold a central position in maintaining homeostasis in the liver as well as in the pathogenesis of acute or chronic liver injury. We showed that MSC-derived PGE_2_ ameliorated macrophage activation-induced inflammatory response. Recent studies have revealed macrophages to be heterogeneous; M2 macrophages protected against exacerbated inflammation and thus limited tissue injury [6]. Several studies have showed the role of MSC on macrophage polarization [19, 20]. Our studies demonstrated the role of MSC-derived PGE_2_ on M2 macrophage induction in ALF thus limiting liver injury, as the increased expression of M2 markers and plasma levels of IL-10. Next, we found that MSC-derived PGE_2_ not only activated STAT6 signaling in BMDM, the classical pathway on M2 macrophage polarization [31], but also enhanced the expression of mTOR signaling substrates, which has shown to control macrophage metabolism and thus to shape their properties [21, 32]. Administration of the mTOR inhibitor or AKT-specific inhibitor significantly inhibited the ability of MSC-derived PGE_2_ on M2 macrophage polarization.

PGE_2_ elicits a wide range of biological effects through its receptors, EP1-EP4 [23]. Our previous study found that MSC-derived PGE_2_ promoted hepatocyte proliferation via EP4. In this study, we administed EP4 antagonist to mice, which significantly inhibited the effects of MSC on attenuating liver inflammation and M2 macrophage polarization. And the administration of an agonist for EP4 has been found to confer protection against IRI in the liver, ischemic heart diseases, and intestinal injury [33–35]. Collectively, MSC-derived PGE_2_ protected against ALF through EP4.

In conclusion, our data confirmed that MSC attenuated ALF dependent on the secretion of PGE_2_. MSC-derived PGE_2_ inhibited TAK1 signaling and NLRP3 inflammasome activation in liver macrophages; meanwhile, MSC-derived PGE_2_ could also induce M2 macrophages to secret anti-inflammatory cytokines, like IL-10 to promote inflammation resolution and limit liver injury through activating STAT6 and mTOR signaling in macrophages, finally to inhibit the liver inflammatory response and hepatocyte apoptosis induced by LPS/D-Gal. All these phenomena were relied on the PGE_2_ receptor, EP4. Inhibiting EP4 significantly alleviated the therapeutic potential of MSC on ALF. This study provided a new therapeutic mechanism of MSC on ALF, which might provide a new direction to enhance the functionality of MSC.

### Supplementary Information

**Additional file 1 Table S1.** Antibodies for immunoblots and immunohistochemistry. **Table S2.** Sequence of primers used in experiments. **Table S3.** Primers of siRNAs. **Figure S1:** Identification of mesenchymal stem cells (MSCs). Flow cytometry analysis of MSCs. PE, phycoerythrin. The immunophenotypic characterization by flow cytometry showed positive stromal marker expression (CD29, CD44, and CD90), but little or no hematopoietic marker expression (CD34 and CD45), which means a high purity after the third passage (Fig. S1).

## Data Availability

The authors confirm that all data underlying the findings are fully available.

## Abbreviations

MSC: Mesenchymal stem cell
ALF: Acute liver failure
PGE_2_: Prostaglandin E_2_
TAK1: TGF-β-activated kinase 1
mTOR: Mechanistic target of rapamycin
TNF: Tumor necrosis factor
IL: Interleukin
ROS: Reactive oxygen species
DAMPs: Damage-associated molecular patterns
DC: Dendritic cells
NLRP3: Nucleotide-binding and oligomerization domain-like receptor 3
ASC: Apoptosis-associated speck-like protein
PAMPs: Pathogen-associated molecular patterns
ALT: Alanine aminotransaminase
AST: Aspartate aminotransferase
BMDM: Bone marrow-derived macrophage
M-CSF: Macrophage colony-stimulating factor
ConA: Concanavalin A
IRI: Ischemia-reperfusion injury
MAPKs: Mitogen-activated protein kinases
NASH: Nonalcoholic steatohepatitis
