# Correction: Mesenchymal stem cell-secreted prostaglandin E2 ameliorates acute liver failure via attenuation of cell death and regulation of macrophage polarization

**DOI:** 10.1186/s13287-023-03570-7

**Published:** 2023-11-23

**Authors:** Jinglin Wang, Yang Liu, Haoran Ding, Xiaolei Shi, Haozhen Ren

**Affiliations:** 1 Department of Hepatobiliary Surgery, Affiliated Drum Tower Hospital of Nanjing University Medical School, Nanjing, Jiangsu Province China; 2 Department of Hepatobiliary Surgery, Nanjing Drum Tower Hospital Clinical College of Nanjing Medical University, Nanjing, Jiangsu Province China; 3 Department of Hepatobiliary Surgery, Nanjing University of Chinese Medicine, Nanjing, Jiangsu Province China

**Correction : Stem Cell Research & Therapy (2021) 12:15** 10.1186/s13287-020-02070-2

Following the publication of this article, the authors regretfully found one errors in the article and would like to make corrections:

The word “PI” mentioned in the article was wrongly described and should be changed to “Cleaved PARP (Asp214)”. PARP has been reported to cleave when cells undergo apoptosis or necrosis in previous studies. Thus, cleavage analysis of this specific protein can be a sensitive tool to detect apoptosis or necrosis. However, the datasheet of Cleaved PARP (Asp214) kit indicated the cleaved PARP serves as a marker of apoptosis. To clear up this confusion, in the Materials and methods section, “TUNEL and PI assay” should be corrected to “TUNEL and Cleaved PARP (Asp214) assay”, “Cell apoptosis and death in the liver were measured using the one-step TUNEL Apoptosis Assay kit and propidium iodide (PI) staining (Beyotime Biotechnology, Shanghai, China) according to the manufacturer’s instructions” should be corrected to “Cell apoptosis in the liver were measured using the one-step TUNEL Apoptosis Assay kit and Cleaved PARP (Asp214) (Cell Signaling Technology, #94885) according to the manufacturer’s instructions”. In the Results section, “TUNEL and PI staining confirmed the hepatocyte necrosis and apoptosis were decreased when infused with MSC, especially in the MSC-COX2(+) group; however, MSC-COX2(−) failed to decrease hepatocyte necrosis and apoptosis (Fig. 1c, d)” should be corrected to “TUNEL and Cleaved PARP (Asp214) staining confirmed the hepatocyte apoptosis were decreased when infused with MSC, especially in the MSC-COX2(+) group; however, MSC-COX2(−) failed to decrease hepatocyte apoptosis (Fig. 1c, d)”.

**Fig. 1:**
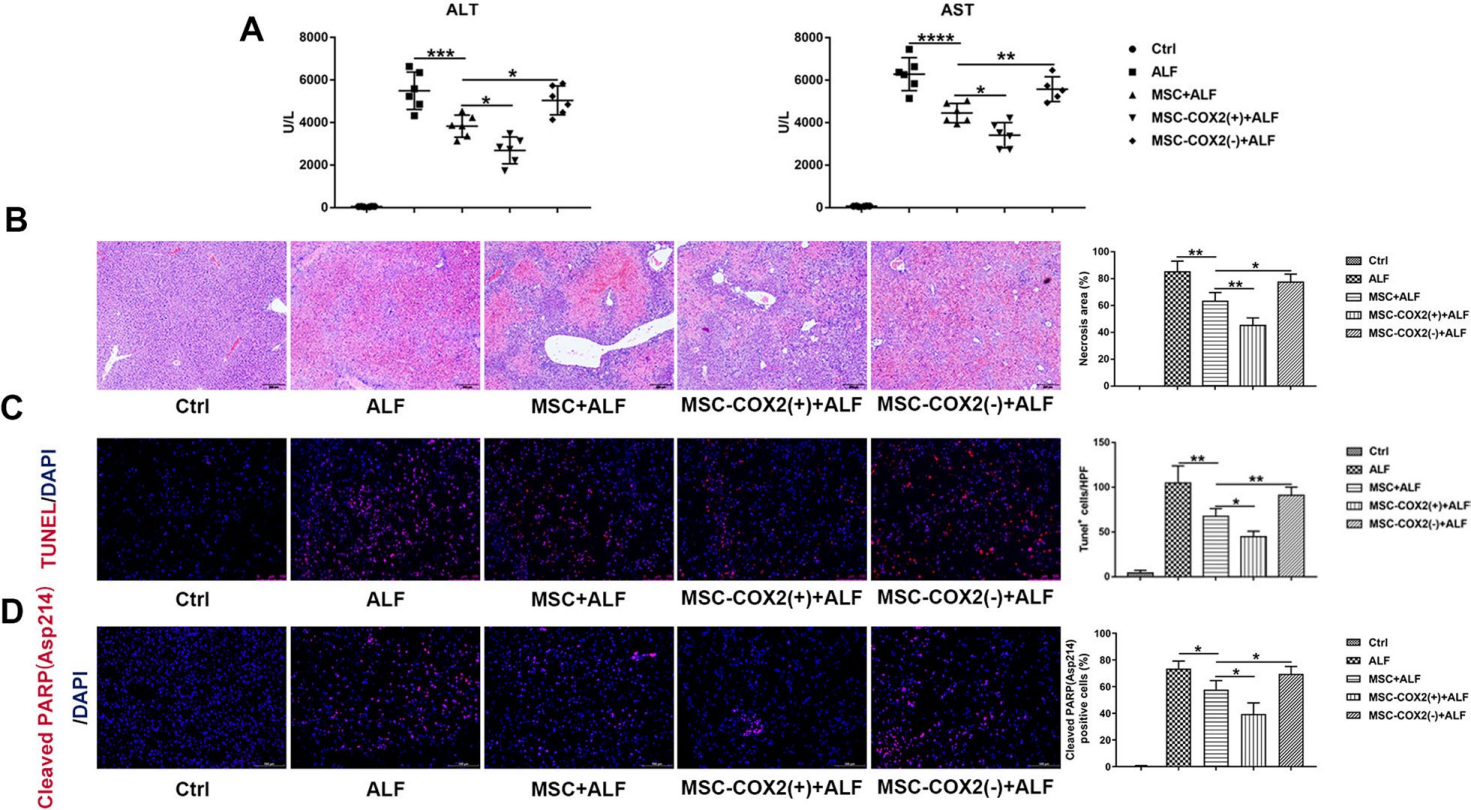
MSC protects LPS/D-Gal-induced liver injury via PGE2. **a** Serum levels of ALT and AST in each group (n = 6).** b** Representative HE-stained liver sections from each group and quantitation of the necrosis area in each group (n = 4). **c** Representative images of TUNEL-stained liver sections from each group and the number of TUNEL-positive cells in each group (n = 4). **d** Representative images of Cleaved PARP (Asp214) staining to indicate apoptosis cells and the number of Cleaved PARP (Asp214) -positive cells in each group (n = 4) (*p < 0.05, **p < 0.01, ***p < 0.001, ****p < 0.0001).

Specifically, we want to make the following changes:

1. Figure 1: in Figure 1d the label of the immunofluorescence staining is mislabeled, which should be Cleaved PARP (Asp214)/DAPI; in the figure legend, change “Representative images of PI staining to indicate necrosis cells and the number of PI-positive cells in each group” to “Representative images of Cleaved PARP (Asp214) staining to indicate apoptosis cells and the number of Cleaved PARP (Asp214) -positive cells in each group”.
2. Figure 2: in Figure 2f the western blots band of c-Jun was wrongly used. The correct image has been replaced.

**Fig. 2:**
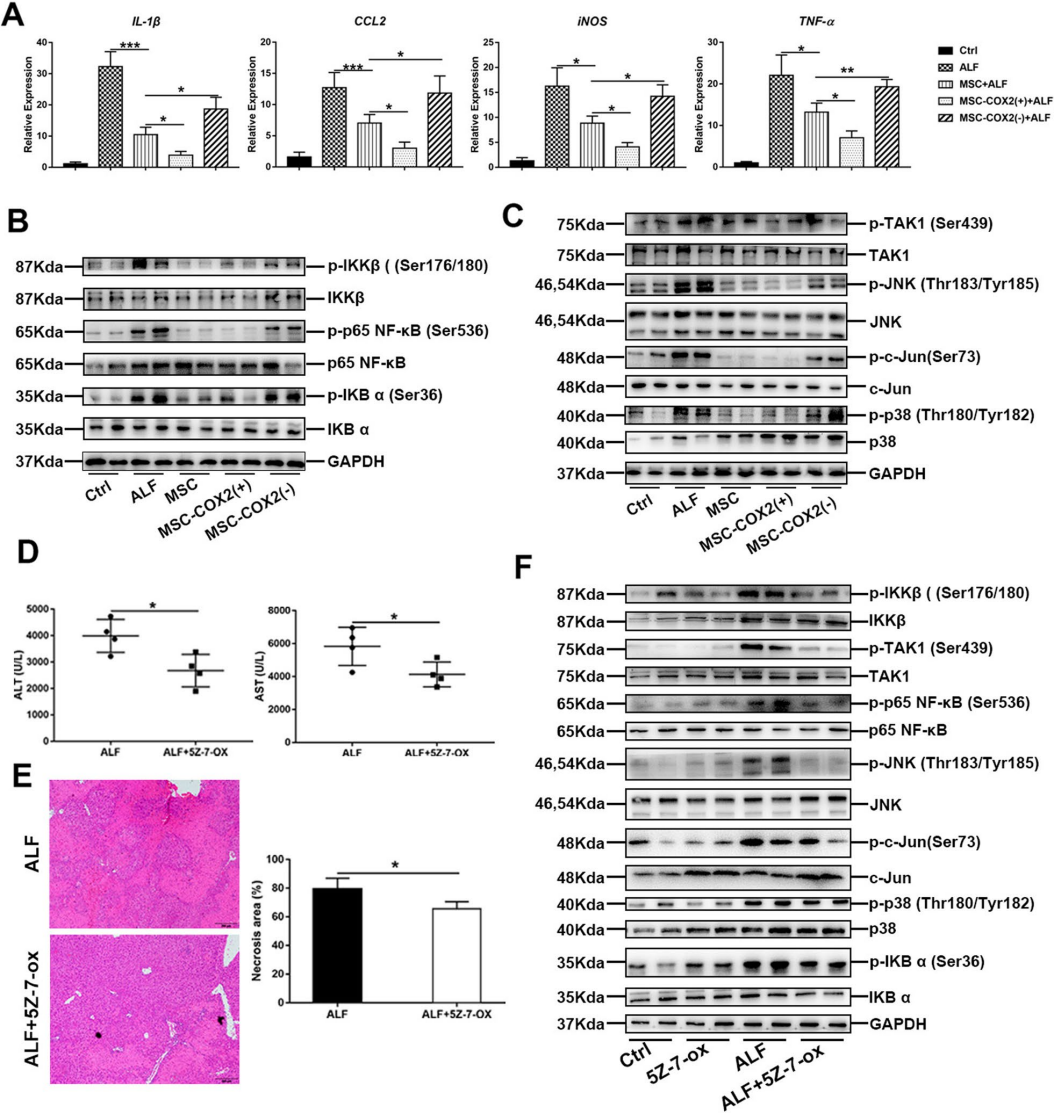
MSC-derived PGE2 restrains inflammatory responses in the liver during LPS/D-Gal-induced ALF. **a** The mRNA levels of pro-inflammatory cytokines (IL-1β, CCL2, iNOS, TNF-α) in the liver in each group (n = 4). **b** The protein expression levels of NF-κB signaling in each group. **c** The protein expression levels of TAK1 signaling in each group. **d** Serum levels of ALT and AST in mice pretreated with TAK1 inhibitor, 5Z-7-ox (n = 4). **e** Representative HE-stained liver sections and quantitation of necrosis area in each group (n = 4). **f** Protein levels of TAK1 and NF-κB signaling in each group (*p < 0.05, **p < 0.01, ***p < 0.001).
3. Figure 7: in Figure 7c the western blots band of p-TAK1 (Ser439) was wrongly used. The correct image has been replaced.

**Fig. 7:**
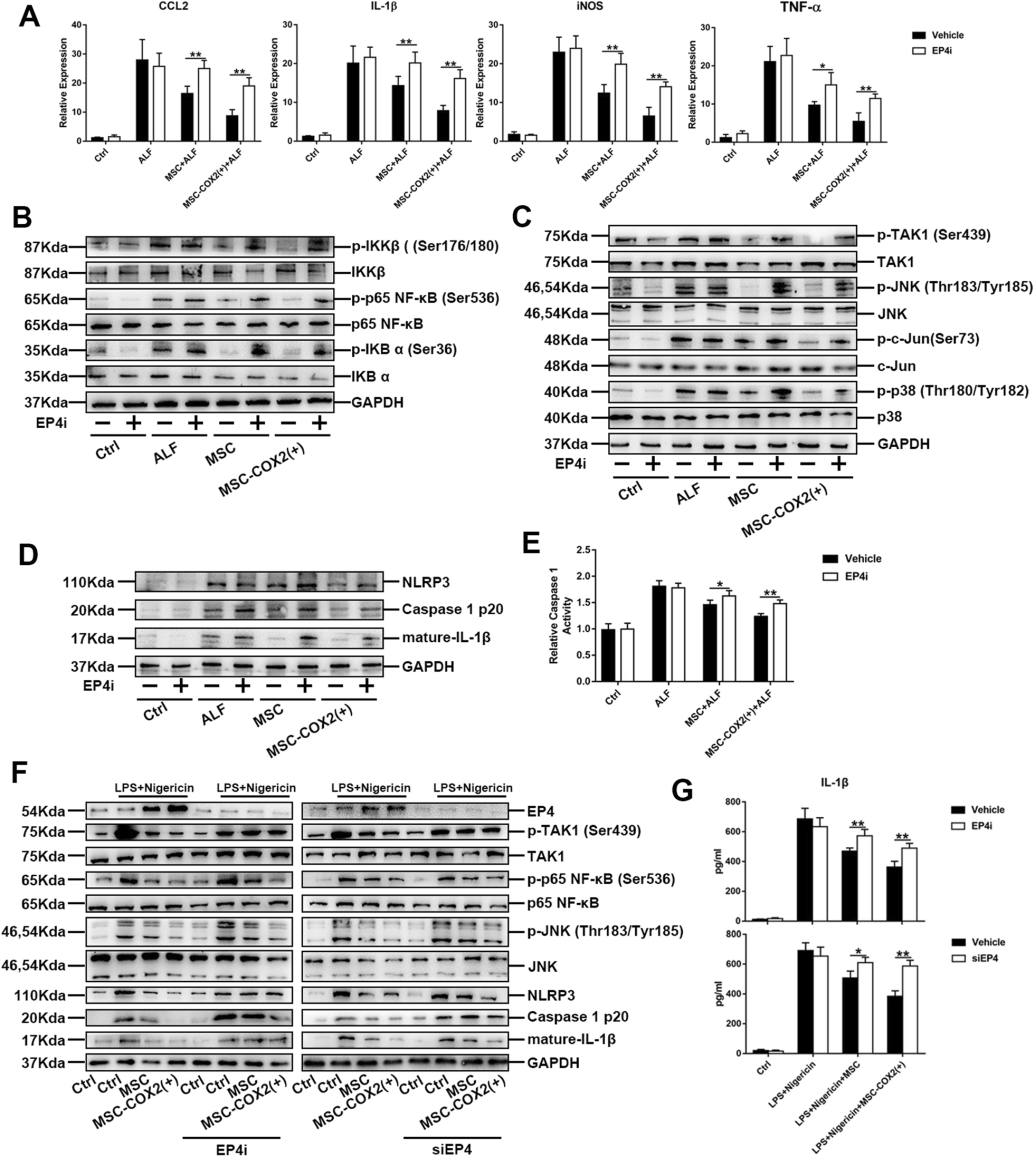
MSC-derived PGE2 protects against liver inflammation via EP4. **a** The mRNA levels of inflammatory cytokines (IL-1β, CCL2, iNOS, TNF-α) in the liver from each group pretreated with EP4 inhibitor (EP4i) (n = 4). **b** The protein expression levels of NF-κB signaling in each group pretreated with EP4i. **c** The protein expression levels of TAK1 signaling in each group pretreated with EP4i. **d** The protein expression levels of NLRP3 inflammasome signaling in each group pretreated with EP4i. **e** Measurement of caspase-1 enzymatic activity in the liver of each group pretreated with EP4i (n = 4). **f** Protein levels of NLRP3 inflammasome and TAK1 signaling in BMDM pretreated with EP4i or knocking down of EP4 via siRNA. **g** The levels of IL-1β in supernatants of BMDM pretreated with EP4i or knocking down of EP4 via siRNA (*p < 0.05, **p < 0.01)
4. Page 4, left column, lines 10, change “Fig. 2e” to “Fig. 2f”.

The corrected figures:

Figures 1, 2 and 7.

These corrections will not affect the result and conclusion of the article. We sincerely apologize for any inconvenience caused.

