# Supplementary material for: Mesenchymal stem cell-secreted prostaglandin E2 ameliorates acute liver failure via attenuation of cell death and regulation of macrophage polarization
Source: Stem Cell Res Ther. 2021 Jan 7;12:15. doi: 10.1186/s13287-020-02070-2 (PMC7792134; doi:10.1186/s13287-020-02070-2)
Supplement: Supplementary file 1 — Additional file 1 Table S1. Antibodies for immunoblots and immunohistochemistry. Table S2. Sequence of primers used in experiments. Table S3. Primers of siRNAs. Figure S1: Identification of mesenchymal stem cells (MSCs). Flow cytometry analysis of MSCs. PE, phycoerythrin. The immunophenotypic characterization by flow cytometry showed positive stromal marker expression (CD29, CD44, and CD90), but little or no hematopoietic marker expression (CD34 and CD45), which means a high purity after the third passage (Fig. S1). [file 13287_2020_2070_MOESM1_ESM.docx]

**Table S1. Antibodies for immunoblots and immunohistochemistry**

| **Antibodies** | **Company** | **Dilution** | **Catalogue number** |
| --- | --- | --- | --- |
| GAPDH | Abcam | 1:10000 | ab181603 |
| p- IKKβ (Ser176/180) | Cell Signaling Technology | 1:1000 | 2697 |
| IKKβ | Cell Signaling Technology | 1:1000 | 8943 |
| p-NF-κB (Ser536) | Cell Signaling Technology | 1:1000 | 3033 |
| NF-κB | Cell Signaling Technology | 1:1000 | 8242 |
| p- IKBα(Ser36) | Abcam | 1:5000 | ab133462 |
| IKBα | Abcam | 1:5000 | ab32518 |
| p-TAK1(Ser439) | Abcam | 1:1000 | ab109404 |
| TAK1 | Abcam | 1:5000 | ab109526 |
| p-JNK (Thr183/Tyr185) | Cell Signaling Technology | 1:1000 | 9251 |
| JNK | Cell Signaling Technology | 1:1000 | 9252 |
| p-c-Jun (Ser73) | Cell Signaling Technology | 1:1000 | 9261 |
| c-Jun | Cell Signaling Technology | 1:1000 | 9165 |
| p-p38 (Thr180/Tyr182) | Cell Signaling Technology | 1:1000 | 4511 |
| p38 | Cell Signaling Technology | 1:1000 | 8690 |
| NLRP3 | Adipogen Life Science | 1:1000 (WB)  1:200 (IHC) | AG-20B-0014 |
| Caspase 1 | Santa Cruz Technology | 1:200 | Sc-398715 |
| IL-1β | Cell Signaling Technology | 1:1000 | 31202 |
| F4/80 | Abcam | 1:200 (IHC) | ab6640 |
| CD206 | Abcam | 1:200 (IHC) | ab64693 |
| p-MTOR (Ser2448) | Cell Signaling Technology | 1:1000 | 5536 |
| MTOR | Cell Signaling Technology | 1:1000 | 2983 |
| p-AKT (Ser473) | Abcam | 1:5000 | ab81283 |
| AKT | Abcam | 1:5000 | ab179463 |
| p-STAT6 (Tyr641) | Abcam | 1:1000 | ab28829 |
| STAT6 | Abcam | 1:2000 | ab32520 |
| p-p70 S6K (Thr389) | Cell Signaling Technology | 1:1000 | 9234 |
| p70 S6K | Cell Signaling Technology | 1:1000 | 2708 |
| p-GSK-3β (Ser9) | Cell Signaling Technology | 1:1000 | 5558 |
| GSK-3β | Cell Signaling Technology | 1:1000 | 12456 |
| EP4 | Santa Cruz Technology | 1:200 | sc-55596 |

**Table S2. Sequence of primers used in experiments**

| **Gene** | **Forward** | **Reverse** |
| --- | --- | --- |
| actin | AGTGTGACGTTGACATCCGTA | GCCAGAGCAGTAATCTCCTTCT |
| Ccl2 | TTAAAAACCTGGATCGGAACCAA | GCATTAGCTTCAGATTTACGGGT |
| IL-1β | GCAACTGTTCCTGAACTCAACT | ATCTTTTGGGGTCCGTCAACT |
| iNOS | ACATCGACCCGTCCACAGTAT | CAGAGGGGTAGGCTTGTCTC |
| TNF-α | GACGTGGAACTGGCAGAAGAG | ACCGCCTGGAGTTCTGGAA |
| Arg1 | CTCCAAGCCAAAGTCCTTAGAG | GGAGCTGTCATTAGGGACATCA |
| Mgl1 | TGCAACAGCTGAGGAAGGACTTGA | AACCAATAGCAGCTGCCTTCATGC |
| Mgl2 | GCATGAAGGCAGCTGCTATTGGTT | TAGGCCCATCCAGCTAAGCACATT |
| Ym1 | CAGGTCTGGCAATTCTTCTGAA | GTCTTGCTCATGTGTGTAAGTGA |
| IL10 | GCTATGCTGCCTGCTCTTACT | CCTGCTGATCCTCATGCCA |
| EP1 | CTTAACCTGAGCCTAGCGGAT | ATGTGCCATTATCGCCTGTTG |
| EP2 | CAGCTCGGTGATGTTCTCGG | GAGCACCAATTCCGTTACCAG |
| EP3 | CCGGAGCACTCTGCTGAAG | CCCCACTAAGTCGGTGAGC |
| EP4 | CCATTCCCGCAGTGATGTTCA | TGCGCGACTTGCACAATACTA |

**Table S3. Primers of siRNAs**

| siEP4 | CCATCGTAGTATTGTGCAA |
| --- | --- |

**
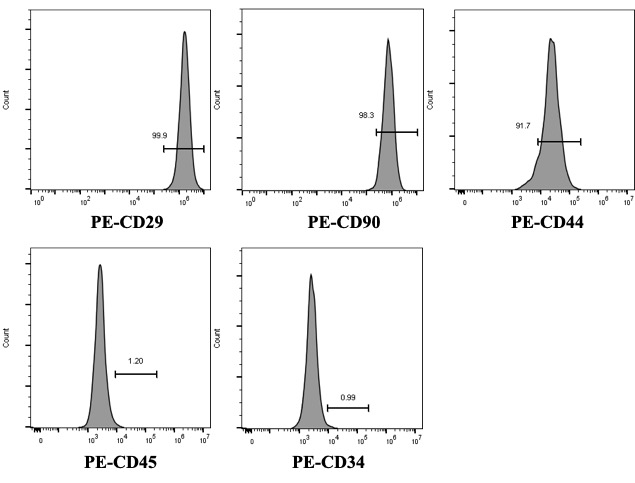
**

**Figure S1:** Identification of mesenchymal stem cells (MSCs). Flow cytometry analysis of MSCs. PE, phycoerythrin.

The immunophenotypic characterization by flow cytometry showed positive stromal marker expression (CD29, CD44, and CD90), but little or no hematopoietic marker expression (CD34 and CD45), which means a high purity after the third passage (Fig. S1).
